# Supplementary material for: The combination of Hebbian and predictive plasticity learns invariant object representations in deep sensory networks
Source: Nat Neurosci. 2023 Oct 12;26(11):1906–15. doi: 10.1038/s41593-023-01460-y (PMC10620089; doi:10.1038/s41593-023-01460-y)
Supplement: Supplementary file 2 — Reporting Summary [file 41593_2023_1460_MOESM2_ESM.pdf]

Corresponding author(s): F. Zenke

Last updated by author(s): Aug 15, 2023

Reporting Summary

Nature Portfolio wishes to improve the reproducibility of the work that we publish. This form provides structure for consistency and transparency in reporting. For further information on Nature Portfolio policies, see our Editorial Policies and the Editorial Policy Checklist.

Statistics

For all statistical analyses, confirm that the following items are present in the figure legend, table legend, main text, or Methods section.

n/a	Confirmed	
☐	☒	The exact sample size (*n*) for each experimental group/condition, given as a discrete number and unit of measurement
☒	☐	A statement on whether measurements were taken from distinct samples or whether the same sample was measured repeatedly
☒	☐	The statistical test(s) used AND whether they are one- or two-sided<br>*Only common tests should be described solely by name; describe more complex techniques in the Methods section.*
☒	☐	A description of all covariates tested
☒	☐	A description of any assumptions or corrections, such as tests of normality and adjustment for multiple comparisons
☐	☒	A full description of the statistical parameters including central tendency (e.g. means) or other basic estimates (e.g. regression coefficient) AND variation (e.g. standard deviation) or associated estimates of uncertainty (e.g. confidence intervals)
☒	☐	For null hypothesis testing, the test statistic (e.g. *F*, *t*, *r*) with confidence intervals, effect sizes, degrees of freedom and *P* value noted<br>*Give P values as exact values whenever suitable.*
☒	☐	For Bayesian analysis, information on the choice of priors and Markov chain Monte Carlo settings
☒	☐	For hierarchical and complex designs, identification of the appropriate level for tests and full reporting of outcomes
☒	☐	Estimates of effect sizes (e.g. Cohen's *d*, Pearson's *r*), indicating how they were calculated

Our web collection on statistics for biologists contains articles on many of the points above.

Software and code

Policy information about availability of computer code

Data collection	All simulations were custom written in Python (v 3.8) using PyTorch (v 1.11.0) and the Lightning framework (v 1.6.1). Spiking network simulations were custom-written in C++ based on the Auryn spiking neural network library (v 0.8.2 commit 36b3c197).<br>The simulation code is available at https://github.com/fmi-basel/latent-predictive-learning
Data analysis	Analysis was performed using Python Jupyter Notebooks (v 1.0.0). The notebooks are available at https://github.com/fmi-basel/latent-predictive-learning/notebooks<br>We used Engauge Digitizer (v12.1) to extract datapoints from plots in published papers.

For manuscripts utilizing custom algorithms or software that are central to the research but not yet described in published literature, software must be made available to editors and reviewers. We strongly encourage code deposition in a community repository (e.g. GitHub). See the Nature Portfolio guidelines for submitting code & software for further information.

Data

Policy information about availability of data

All manuscripts must include a data availability statement. This statement should provide the following information, where applicable:

- Accession codes, unique identifiers, or web links for publicly available datasets
- A description of any restrictions on data availability
- For clinical datasets or third party data, please ensure that the statement adheres to our policy

The deep learning tasks used the MNIST, STL-10, 3D Shapes, and CIFAR-10 datasets, typically available through all major machine learning libraries. The original releases for these datasets can be found at http://yann.lecun.com/exdb/mnist/, http://ai.stanford.edu/%7Eacoates/stl10/, https://github.com/deepmind/3d-shapes/, and https://www.cs.toronto.edu/~kriz/cifar.html respectively.

Human research participants

Policy information about studies involving human research participants and Sex and Gender in Research.

Reporting on sex and gender	N/A
Population characteristics	N/A
Recruitment	N/A
Ethics oversight	N/A

Note that full information on the approval of the study protocol must also be provided in the manuscript.

Field-specific reporting

Please select the one below that is the best fit for your research. If you are not sure, read the appropriate sections before making your selection.

☒ Life sciences ☐ Behavioural & social sciences ☐ Ecological, evolutionary & environmental sciences

For a reference copy of the document with all sections, see nature.com/documents/nr-reporting-summary-flat.pdf

Life sciences study design

All studies must disclose on these points even when the disclosure is negative.

Sample size	We used no statistical tests in this study, nor did we use statistical methods to predetermine sample sizes. This article is a computational study; sample sizes were selected to make the effects unambiguous. For the deep neural network simulations in Figs. 3 and 4, the sample size was n=4, and we displayed standard errors of the mean. We performed spiking simulations with stochastic Poisson input and random sparse connectivity using the same random seed for each setting in Fig. 5. We computed the summary statistics in Fig. 5c-e from the resulting sparse input connectivity matrix comprising 5082 synaptic connections.
Data exclusions	No data were excluded from the analysis.
Replication	We used n=4 random initializations of the deep neural network model. We also used random augmentations and ordering to present the training examples and ensured the robustness of the results when selectively varying simulation parameters. However, all reported results were consistent across many simulations. All replication attempts were successful, and our publicly available code can be tested and scrutinized for replicability by others.
Randomization	Randomization was irrelevant to our study because it did not involve group allocation. We had no experimental samples/organisms/ participants in our study.
Blinding	Blinding was not relevant because our study did not involve group allocation.

Reporting for specific materials, systems and methods

We require information from authors about some types of materials, experimental systems and methods used in many studies. Here, indicate whether each material, system or method listed is relevant to your study. If you are not sure if a list item applies to your research, read the appropriate section before selecting a response.

Materials & experimental systems

n/a	Involved in the study
☒	☐ Antibodies
☒	☐ Eukaryotic cell lines
☒	☐ Palaeontology and archaeology
☒	☐ Animals and other organisms
☒	☐ Clinical data
☒	☐ Dual use research of concern

Methods

n/a	Involved in the study
☒	☐ ChIP-seq
☒	☐ Flow cytometry
☒	☐ MRI-based neuroimaging
